# Layer- and Cell Type-Specific Modulation of Excitatory Neuronal Activity in the Neocortex

**DOI:** 10.3389/fnana.2018.00001

**Published:** 2018-01-30

**Authors:** Gabriele Radnikow, Dirk Feldmeyer

**Affiliations:** ^1^Research Centre Jülich, Institute of Neuroscience and Medicine, INM-10, Jülich, Germany; ^2^Department of Psychiatry, Psychotherapy and Psychosomatics, Medical School, RWTH Aachen University, Aachen, Germany; ^3^Jülich-Aachen Research Alliance – Translational Brain Medicine, Jülich, Germany

**Keywords:** barrel cortex, cortical layers, neuromodulation, acetylcholine, adenosine, dopamine, orexin

## Abstract

From an anatomical point of view the neocortex is subdivided into up to six layers depending on the cortical area. This subdivision has been described already by Meynert and Brodmann in the late 19/early 20. century and is mainly based on cytoarchitectonic features such as the size and location of the pyramidal cell bodies. Hence, cortical lamination is originally an anatomical concept based on the distribution of excitatory neuron. However, it has become apparent in recent years that apart from the layer-specific differences in morphological features, many functional properties of neurons are also dependent on cortical layer or cell type. Such functional differences include changes in neuronal excitability and synaptic activity by neuromodulatory transmitters. Many of these neuromodulators are released from axonal afferents from subcortical brain regions while others are released intrinsically. In this review we aim to describe layer- and cell-type specific differences in the effects of neuromodulator receptors in excitatory neurons in layers 2–6 of different cortical areas. We will focus on the neuromodulator systems using adenosine, acetylcholine, dopamine, and orexin/hypocretin as examples because these neuromodulator systems show important differences in receptor type and distribution, mode of release and functional mechanisms and effects. We try to summarize how layer- and cell type-specific neuromodulation may affect synaptic signaling in cortical microcircuits.

## Introduction

**FIGURE 1:**
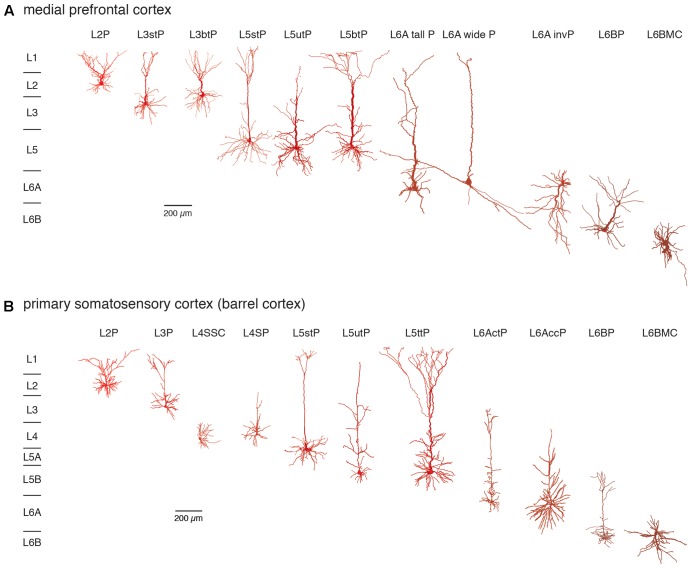
Excitatory neuron types in layers 2–6 of the **(A)** medial prefrontal and **(B)** primary somatosensory (barrel) cortex. Different excitatory neuron types in cortical layers 2–6 (L2–L6) of rat mPFC and S1 barrel cortex are shown. Most neuron types are pyramidal cells with apical dendrites of different shape and length with the exception of spiny stellate cells in layer 4 and multipolar neurons in layer 6B. Somatodendritic domains are shown in different shades of red, with bright red indicating superficial and dark red deep layers. Note that the diversity of excitatory neurons is much higher than that shown here and that even between e.g., sensory cortices different pyramidal cell types can be found. L2P: L2 pyramidal cell; L3stP: L3 slender-tufted pyramidal cell, L3btP: L3 broad-tufted pyramidal cell; L3P: L3 pyramidal cell; L4SSC: L4 spiny stellate cell; L4SP: L4 star pyramidal cell; L5stP: L5 slender-tufted pyramidal cell (with strong axonal projections to layer 2 and 3); L5utP: L5 untufted pyramidal cell; L5btP: L5 broad-tufted pyramidal cell and L5ttP: L5 thick-tufted pyramidal cell (both of which project mainly to subcortical targets); L6A tall P: L6A tall pyramidal cell; L6A wide P: L6A wide pyramidal cell; L6A invP: L6A inverted pyramidal cell; L6AccP: L6A corticocortical pyramidal cell; L6ActP: L6A corticothalamic pyramidal cellL6AP: L6BP L6B pyramidal cell; L6BMC: L6B multipolar cell. This terminology will be used throughout the remainder of the text.

The notion that the neocortex is subdivided into six different laminae was first introduced around the middle of the 19th century and primarily based on its cytoarchitecture, i.e., the distribution and size of pyramidal cell bodies (Meynert, 1867; Brodmann, 1909) and myeloarchitecture, i.e., the projection pattern of long range, intracortical axon (Baillarger, 1840; Vogt, 1906; see also von Economo, 1929). **Figure 1** gives an overview of neocortical excitatory neuron types in the different layers of two cortical areas, the medial prefrontal and the primary somatosensory cortex (for an in-depth review of cortical lamination and excitatory neuron types see also Narayanan et al., 2017).

It is apparent that excitatory neuron size and shape varies markedly within and between layers but also between different brain regions. We will use the terminology presented in this figure throughout the remainder of this review.

Thus, originally cortical layers were defined by anatomical features. However, it has been demonstrated that a number of genes (in particular those that encode transcription factors or proteins involved in synaptic signaling) exhibit a clear patterned expression delineating cortical layers. Furthermore, neuronal cell types with different axonal projection patterns showed a differential gene expression suggesting that cortical lamination is not a just an anatomical concept but reflects the segregation of different neuron types into different cortical layers. Of the large number of layer- and neuron-specific genetic markers found in rodents a many have also been identified in primates (Hattox and Nelson, 2007; Belgard et al., 2011; Bernard et al., 2012; Hawrylycz et al., 2012; Lodato and Arlotta, 2015; Molyneaux et al., 2015; Zeisel et al., 2015; Tasic et al., 2016; Lein et al., 2017; Luo et al., 2017).

At a functional level, cell type-specific properties of excitatory neurons including intrinsic properties such as the passive electrical properties, their action potential (AP) firing pattern, their synaptic properties and protein/gene expression pattern have not been comprehensively studied. Only in recent years high-resolution descriptions of the different, in particular long-range axonal projection patterns of excitatory neocortical neurons have become available (Morishima et al., 2011; Oberlaender et al., 2012; Narayanan et al., 2015). A correlation of the morphological, electrophysiological and expression data to unequivocally identify excitatory neocortical neuron types has not been attempted so far and a comprehensive picture of the synaptic properties of the different identified neuronal cell types has not yet emerged.

The function of the neuronal cell types in the different cortical layers is also affected by neuromodulatory transmitters. These neuromodulators regulate the excitability of a neuron (i.e., the probability and efficacy of AP generation and propagation) by affecting ion channels (mostly different K^+^ channels types) and the efficacy and reliability of synaptic transmission via changes in the presynaptic Ca^2+^ channel activity. Most neuromodulator receptors are coupled to different types of G-proteins and act therefore on a significantly slower time scale than ligand-gated ion channels; however, the affinity of G-protein coupled neuromodulator receptors is several orders of magnitude higher than that of ligand-gated channels. While direct synaptic transmission is ‘wired,’ i.e., occurs only at synaptic contacts, the release of neuromodulators is less directed and is often mediated by so-calledd ‘volume transmission’, i.e., by diffusion of the neuromodulator over a larger distance, which will affect not only one neuron but rather neuron ensembles in the vicinity of the neuromodulator release site (Zoli et al., 1999; Taber and Hurley, 2014; Badin et al., 2016). There are many different neuromodulator types which are either released from small groups of subcortical neurons that send their axon into the neocortex (such as cholinergic afferents form the basal forebrain) or are produced intracortically (such as adenosine). While it has been shown that differences in neuromodulator receptor expression exist, studies addressing a layer- and neuronal cell-type their layer-specific action are just beginning to emerge.

In this review we will focus on four different types of neuromodulators that differ in many aspects, including their mode of release, mechanism of action and target structures. First, we will discuss the nucleotide adenosine which is released in a non-vesicular fashion. Second, we will describe the cholinergic system which is noteworthy because it acts on two different neuromodulatory systems, the fast nicotinic acetylcholine (ACh) receptor channels (nAChRs) and the slow, G-protein coupled muscarinic ACh receptors (mAChRs). Third, we will address the dopaminergic system as an example of neuromodulation by a monoamine and finally peptidergic modulation by orexin/hypocretin. The underlying biophysical and biochemical mechanisms of the function of these neuromodulator systems will only be discussed in the context of their effects in different cortical layers and on different neuron types. We will mainly concentrate here on data from functional, mostly electrophysiological studies which allow a cell-specific examination of neuromodulator action and its underlying mechanisms such as the coupled G-Protein type and ion channel types activated via intracellular enzyme cascades as well as the coupled ionotropic nAChR channel subtypes. However, this data will be put in context with earlier *in situ* hybridisation, immunohistochemical, receptor autoradiography and electronmicroscopy studies whenever necessary or possible.

## Brief Overview of G-Protein Signaling Mechanisms

The effects of most of the neuromodulator systems reviewed here are mediated via G-protein coupled receptors (GPCRs). G-proteins can be broadly subdivided into four different groups with different signaling pathways, namely the G_i/o_-, G_s_-, and G_q/11_- and G_12/13_ G-protein families (for a review see Oldham and Hamm, 2008). Neuromodulator receptors can be coupled to the first three G-protein types but not to G_12/13_ proteins which have mainly cytoskeletal function by regulating actin dynamics.

**FIGURE 2:**
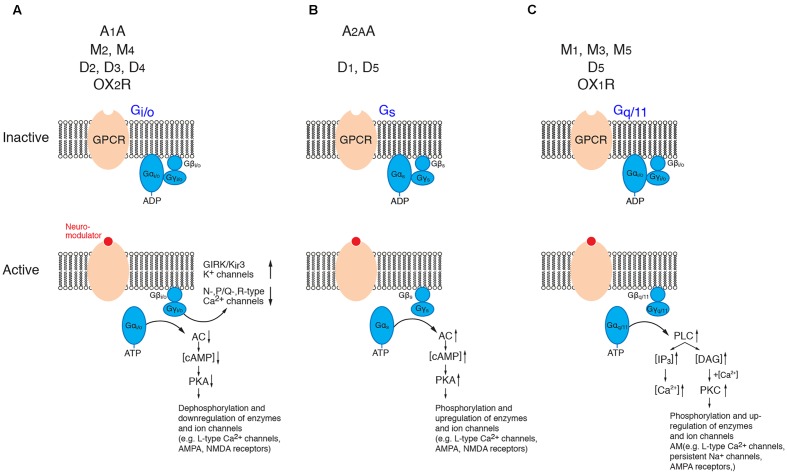
Signaling cascades of neuromodulator-coupled G-proteins. Signaling pathways of the G-protein coupled receptors (GPCRs) discussed in this review (top row). **(A)** G_i/o_ signaling, **(B)** G_s_ signaling and **(C)** G_q/11_ signaling pathways. Signaling occurs via the dissociated and phosphorylated Gα subunit or via direct interaction between the βγ subunit complex and the effector (K^+^- and Ca^2+^ channels). See text for details. It should be noted that the downstream signaling pathways of PKA, PKC and PLC are significantly more diverse than shown here. (AC, adenylate cyclase; ADP, adenosine diphosphate; ATP, adenosine trisphosphate; cAMP, cyclic adenosine monophosphate; DAG, diacylglycerol; GIRK, G-protein coupled, inwardly rectifying K^+^ channel; IP_3_, inositol trisphosphate; PKA, phosphokinase A; PKC, phosphokinase C; PLC; phospholipase C). For the abbreviation of receptor subtypes see text.

G-proteins are membrane-bound proteins consisting of three different subunits, the large α- and the smaller β- and γ-subunits, the latter of which form a dimeric β/γ-complex. In its inactive form, the G-protein α-subunit binds GDP which upon activation of the GPCR is exchanged for GTP. This results in a dissociation of the α-subunit from the β/γ-complex and the receptor molecule and in turn initiates many different signaling cascades of which only a few are shown in **Figure 2**. The α-subunit affects downstream second messenger cascades. Basically, the G_i/o_ α-subunit inhibits while the G_s_ α-subunit activates the adenylate cyclase (AC) – phosphokinase A (PKA) pathway that is involved in the phosphorylation of target enzymes and ion channels such as voltage-gated L-type Ca^2+^ channels (Ca_v_1) (Dittmer et al., 2014; Murphy et al., 2014). The G_q_ α-subunit activates phospholipidase C (PLC) which hydrolyses membrane-bound phosphatidylinositol 4,5-bisphosphate to inositol trisphosphate (IP_3_) and diacyl glycerol (DAG). IP_3_ will open IP_3_-sensitive Ca^2+^ channels of the endoplasmic reticulum and cause intracellular Ca^2+^ release. DAG, on the other hand, in combination with an increase in intracellular Ca^2+^ activates protein kinase C (PKC) which leads to the activation of many downstream signaling cascade including, e.g., an increased neuronal excitability by up regulating a persistent Na current (Astman et al., 1998) and an enhancement of synaptic transmission via the phosphorylation of AMPA-type glutamate receptors (Lee et al., 2000; McDonald et al., 2001).

In addition to its α-subunit mediated effects, β/γ-subunit complex of G_i/o_ proteins affects the G-protein coupled, inwardly rectifying K^+^-channels (GIRK or K_ir_3) (for reviews see Doupnik, 2008; Lüscher and Slesinger, 2010; Dascal and Kahanovitch, 2015) and voltage-gated Ca^2+^ channels of the N-, P/Q and R-type (Ca_v_2.2, Ca_v_2.1, Ca_v_2.3) (Zamponi et al., 2015; Huang and Zamponi, 2017). The modulation via the β/γ-subunit complex is direct (i.e., not via a second messenger pathway) and thus significantly faster (<1 s) than that initiated by α-subunits. It is a so-called membrane-delimited step because the β/γ-subunit complex diffuses over a short distance within the cell membrane (for reviews see Doupnik, 2008; Lüscher and Slesinger, 2010; Dascal and Kahanovitch, 2015; Zamponi et al., 2015; Huang and Zamponi, 2017).

## Adenosine Receptors

Adenosine is an almost ubiquitous endogenous neuromodulator and has been implicated in sleep homoeostasis and energy metabolism of neurons (Ribeiro et al., 2002; Porkka-Heiskanen and Kalinchuk, 2011). It is generated during high neuronal activity, e.g., by ATP-dependent ion transporters that are necessary to maintain intracellular ionic homeostasis (for reviews see Fredholm et al., 2005; Sebastião and Ribeiro, 2009). Adenosine is a metabolite of the intracellular ATP degradation; it is transported into the extracellular space by nucleoside transporters which are located in all cellular compartments of a neuron, i.e., dendrites, soma and axon. In addition, membrane bound ATPase (EctoATPase) can catalyze the formation of adenosine extracellularly by degrading ATP that diffused from the cytoplasm of neurons and glia in the perisynaptic space. Thus, in contrast to the other neuromodulator systems discussed below, adenosine is not a classical neurotransmitter because it is not stored in synaptic vesicles from which it is released.

Of the four different adenosine receptor subtypes that exist, i.e., the A_1_, A_2A_, A_2B_, and A_3_ receptors, only the A_1_ and A_2A_ adenosine receptors (A_1_AR and A_2A_AR) are highly expressed in the CNS. Both have high but different adenosine affinities, activate either G_i/o_ (A_1_AR) or G_s_ (A_2A_AR) proteins and have opposite effects on synaptic transmission (Fredholm et al., 2001, 2005, 2011; Sebastião and Ribeiro, 2009; Chen et al., 2014). They show a differential and partly complementary distribution in different brain regions (Fredholm et al., 2001; Ribeiro et al., 2002). Autoradiography studies demonstrated that the A_1_AR mRNA expression is abundant in the neocortex, cerebellum, hippocampus and the dorsal horn of the spinal cord and is enriched at synaptic sites; no apparent layer-specificity was found (Cremer et al., 2011). On the other hand, A_2A_AR mRNA is strongly expressed in striato-pallidal GABAergic neurons and the olfactory bulb but only weakly so in the neocortex; only a suppressive effect of A_1_AR on inhibitory transmission in layer 2/3 has been reported (Bannon et al., 2014). Therefore, only the laminar- and cell-specific effects of A_1_ARs will be discussed below. It should be noted that adenosine receptors are not only expressed in neurons but also in glial cells such as astrocytes and microglia.

Adenosine binding to A_1_ARs activates G_i/o_ proteins. This results in an increased open probability of K_ir_3 channels and a decrease in the open probability of Ca^2+^ channels via the fast, direct interaction with the Gβ/γ subunit complex (see **Figure 2A**). The activation of K_ir_3 channels by adenosine will result in a hyperpolarisation of the resting membrane potential in the majority of excitatory neurons but was not found in inhibitory neocortical interneurons (van Aerde et al., 2015).

**FIGURE 3:**
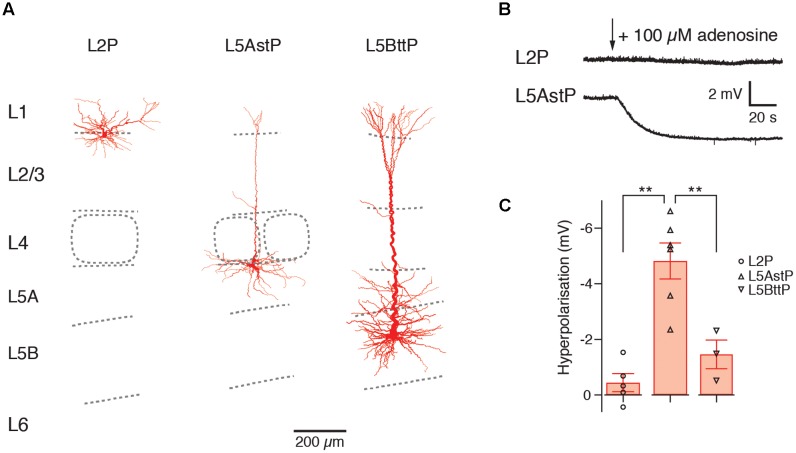
Layer- and cell type-specific difference in the adenosine response in the somatosensory barrel cortex. Significant layer- and cell type-specific differences in the adenosine response in cortical layers 2, 5A and 5B **(A)** Reconstructions of L2, slender-tufted L5A and thick-tufted L5B pyramidal cells in the in the somatosensory barrel cortex. **(B)** Voltage response to adenosine application in L2 and L5A pyramidal cells; L2 pyramidal cells are almost unresponsive to adenosine. **(C)** Comparison of the adenosine response in layers 2, 5A and 5B showing layer-specific differences in the amplitude of the hyperpolarization. This may indicate cell type-specific differences in the density of adenosine A_1_ARs. L3, L4, and L6 pyramidal cells show also a hyperpolarizing response to adenosine (not shown). Modified from van Aerde et al. (2015).

The A_1_AR-mediated hyperpolarizing response shows clear and significant layer- and cell-dependent differences in amplitude. Notably, in both prefrontal cortex (PFC) and primary somatosensory (S1) barrel cortex, L2 pyramidal cells showed no adenosine-induced hyperpolarisation at all (van Aerde et al., 2015), thereby defining this layer by its functional properties. It was found that PFC L3 pyramidal cells displayed mixed and cell type-specific adenosine effects (as defined by their morphological and electrophysiological properties). L3 pyramidal cells that showed a regular firing pattern (about a quarter of the total) were unresponsive to adenosine, with all others showing a weak to strong hyperpolarisation. In layer 4 of the S1 barrel cortex, all excitatory neurons were hyperpolarised by adenosine. L5 pyramidal cells showed also a hyperpolarisation in response to A_1_AR activation. However, the response amplitude was significantly larger in slender-tufted (L5A) pyramidal cells than thick-tufted (L5B) pyramidal cells and largest in PFC L5 pyramidal cells with long basal dendrites (see **Figure 3** and van Aerde et al., 2015). It has been demonstrated that thick-tufted pyramidal cells project mainly sub-cortically while slender-tufted pyramidal cells show dense axonal collaterals in superficial layers 2 and 3 (Molnár and Cheung, 2006; Oberlaender et al., 2011) suggesting a target-specificity in the A_1_AR density in these neuron types. This finding was comparable for both S1 barrel cortex and PFC indicating that the A_1_AR response is conserved between different cortical areas.

**FIGURE 4:**
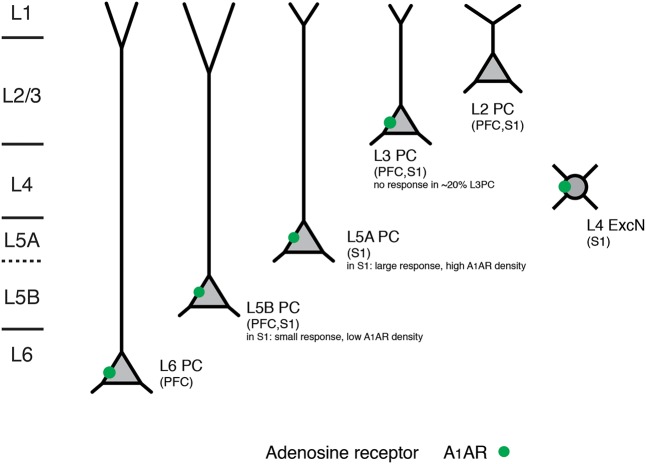
Layer- and cell type-specific A1 adenosine receptor distribution in the prefrontal and primary somatosensory barrel cortex. Adenosine receptors on excitatory neocortical neurons can be found in cortical layers 3–6. Note that in both prefrontal and somatosensory cortex L2 (upper L2/3) pyramidal cells with broad apical tufts were unresponsive to adenosine suggesting no or a very low expression of A_1_ adenosine receptors. In layer 5 of somatosensory cortex two pyramidal cell types showed marked differences in their adenosine response that was correlated with their morphology and laminar location; such a clear difference was not found for the prefrontal cortex. All data are from rat.

PFC L6 pyramidal neurons showed an adenosine response that was comparable to that of slender-tufted L5 pyramidal neurons. In addition, A_1_AR activation decreases thalamocortical excitation of GABAergic interneurons and excitatory neurons in the neocortex (Fontanez and Porter, 2006). In contrast to excitatory neurons, neocortical GABAergic interneurons did not respond to adenosine application (van Aerde et al., 2015). A summary of the layer- and neuronal cell-type specific distribution of A_1_ARs is shown in **Figure 4** and **Table 1**.

**Table 1:** Summary of adenosine receptor, muscarinic and nicotinic ACh receptor, dopamine receptor and orexin receptor effects with respect to cortical layer and cell type.

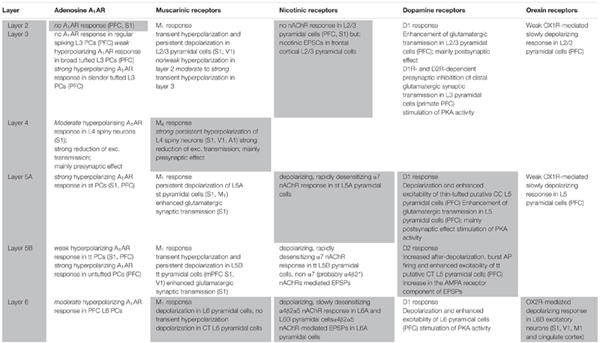

*The table lists the distribution of the neuromodulator receptor subtypes with respect to cortical layers. Technical datails, cortical area and cell-type specificity is given in brackets. Gray shading indicates layer- and cell-specific differences.*

Adenosine also affects excitatory synaptic transmission by causing a reduction in the release probability as shown by a decrease in the amplitude of EPSPs and an increase in the failure rate, variability and paired pulse ratio. This is likely due to a reduced Ca^2+^ channel activity at the presynaptic terminal and has been found for intralaminar L2/3, L4 and L5 and translaminar L4-L2/3 connections (Fontanez and Porter, 2006; Kerr et al., 2013; Bannon et al., 2014; van Aerde et al., 2015; Qi et al., 2016). The synaptic adenosine effect is most likely mediated by a reduction in the open probability of presynaptic Ca^2+^ channels involved in triggering the release of neurotransmitters and is already apparent at low endogenous adenosine concentrations (∼1–2 μM). This in line with the finding that A_1_ARs are predominantly found at synaptic sites (as found in the hippocampus; Rebola et al., 2003) and less so in the dendrites and cell bodies suggesting that the synaptic effect of adenosine is the most prominent and important one.

## Acetylcholine Receptors

Acetylcholine plays a prominent role in arousal, vigilance and attention (for reviews see Hasselmo and Sarter, 2011; Ma et al., 2017). In contrast to adenosine-mediated neuromodulation, acetylcholine (ACh) is released from boutons of axons that originate mainly from neurons in the nucleus basalis of Meynert in the basal forebrain (Mesulam et al., 1983a,b; Yeomans, 2012; Zaborszky et al., 2015). Cholinergic afferents are distributed at very high density throughout all layers of the neocortex, with particularly high axonal bouton densities in layers 6, 5 and 1 (Eckenstein et al., 1988; Henny and Jones, 2008; Kalmbach et al., 2012). ACh may also be (co-)released intracortically from a group of bipolar or fusiform GABAergic interneurons [probably vasoactive intestinal peptide (VIP)-positive interneurons] together with the inhibitory transmitter GABA (Parnavelas et al., 1986; Eckenstein et al., 1988; Umbriaco et al., 1994; von Engelhardt et al., 2007). It has been proposed that most of the intracortical ACh is not released at synaptic contacts proper but rather diffusely into the extracellular space, a mechanism termed ‘volume transmission’ (Descarries et al., 1997; Sarter et al., 2009). However, the presence of intracortical cholinergic synapses has been verified both ultrastructurally (Umbriaco et al., 1994; Turrini et al., 2001; Takács et al., 2013) and functionally (Bennett et al., 2012; Hedrick and Waters, 2015; Hay et al., 2016) for L5 and L6 pyramidal cells as well as for interneurons in layer 1 (Arroyo et al., 2012; Bennett et al., 2012).

The effects of ACh in the neocortex are mediated by two different types of receptors, the G-protein-coupled muscarinic AChRs (mAChRs) and the ionotropic nicotinic AChRs (nAChRs). Both receptor types show cortical layer-specific distributions and effects. These will be discussed separately below.

## Muscarinic Receptors

Muscarinic AChRs (mAChRs) fall into two different subgroups, the M_1_- and the M_2_-type receptors. M_1_-type receptors comprise M_1_, M_3_ and M_5_ mAChRs that are coupled to G_q/11_ proteins. Following ACh binding, the Gα_q/11_ subunit enhances PLC activity resulting in the production of IP_3_ and subsequent Ca^2+^ release from intracellular stores and DAG which activates PKC (see **Figure 2C**). M_2_ and M_4_ mAChRs belong to the M_2_-type receptors that are coupled to G_i/o_ proteins (**Figure 2A**) which inhibit the cyclic adenosine monophosphate (cAMP) signaling pathway by blocking AC and in turn decreases the intracellular cAMP concentration and the PKA activity. This will result in a dephosphorylation of K^+^, Na^+^ and Ca^2+^ and ionotropic GABA and glutamate channels (for reviews see Caulfield and Birdsall, 1998; Thiele, 2013; Muñoz and Rudy, 2014).

The M_1_, M_2_, and M_4_ mAChRs are expressed in the neocortex with the M_1_ receptor (M_1_R) being the most abundant. M_1_Rs show a strong immunoreactivity in layers 2/3 and 6 and a moderate one in layer 5 in both rodent and primate neocortex. Immunoreactivity is associated with both presynaptic axonal boutons and postsynaptic dendritic spines. In contrast, M_2_R expression was found to be high in layer 4 and 5 and only moderate in layer 6. M_4_R mAChRs on the other hand were only weakly expressed in neocortical layer 4 and some L5 neurons (Levey et al., 1991; Mrzljak et al., 1993; for reviews see Brown, 2010; Thiele, 2013). This suggests marked differences in the response to ACh release in different cortical layers and neuron types.

Application of ACh has been shown to induce long-lasting depolarisations of large neocortical pyramidal neurons (McCormick and Prince, 1986). This has lead to the suggestion that ACh mediates an overall increase in cortical excitability. However, recent studies have revealed a more complex picture by demonstrating that excitatory neuron types in different neocortical layers can be distinguished on the basis of their ACh response amplitude and shape.

**FIGURE 5:**
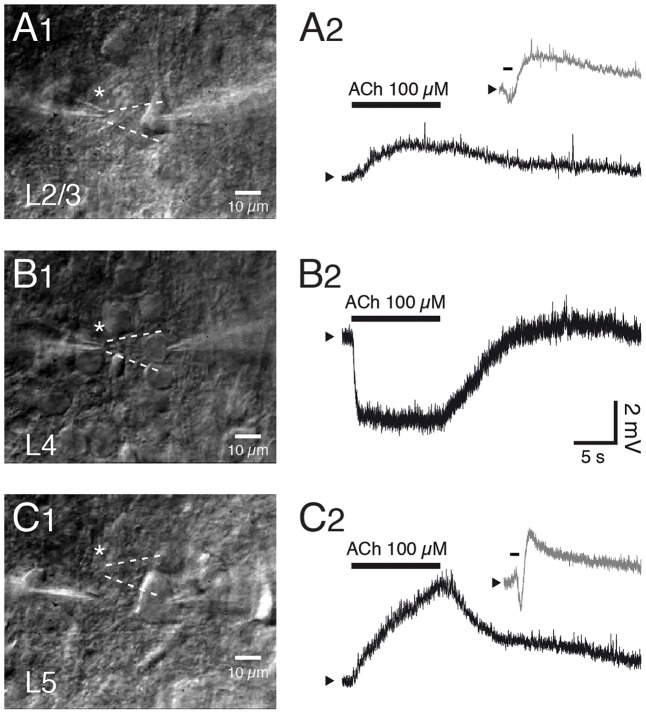
Layer- and cell type-specific muscarinic effects of acetylcholine in the somatosensory barrel cortex. Layer–specific response of excitatory neurons in S1 barrel cortex to rapid application of ACh. **(A1–C1)** Differential interference contrast images of the recorded neurons in layers 2/3, 4 and 5; the profile of the solution ejected by the puff pipette is outlined in white dotted lines. **(A2–C2)** Example responses of L2/3, L4 and L5 excitatory neurons to puff application of ACh. Pyramidal cells in layer 2/3 **(A)** and 5 **(C)** show a depolarization in response to ACh (duration indicated by bar) that is sometimes preceded by a transient hyperpolarization (gray trace in inset). In contrast, all L4 excitatory neurons show a persistent and monophasic hyperpolarizing ACh response.

Overall, a mAChR response was more common and larger in pyramidal cells located in infragranular than in supragranular layers (McCormick and Prince, 1986; Hedrick and Waters, 2015). Most L2/3 pyramidal cells respond to ACh application with a sustained depolarization while a minor fraction of mostly deep L2/3 pyramidal cells respond with an initial small and transient hyperpolarization followed by a sustained depolarisation. Both the transient hyper- and tonic depolarising responses are exclusively mediated by M_1_Rs acting via different K^+^ channel types (see below) and have been observed in PFC, S1 and V1 excitatory neurons (Gulledge and Kawaguchi, 2007; Eggermann and Feldmeyer, 2009; see **Figures 5A1,A2,C1,C2**).

In marked contrast, excitatory neurons in layer 4 of sensory cortices are strongly and persistently hyperpolarised by ACh (**Figures 5B1,B2**). This is due to an increase in the open probability of K_ir_3 channels mediated by M_4_ mAChR activation. The response is similar in L4 excitatory neurons of different sensory cortices, i.e., the primary auditory, S1 and V1 cortex suggesting that the M_4_ AChR response is conserved in sensory cortices. Furthermore, the M_4_ AChRs cause also a suppression of the neurotransmitter release probability at excitatory L4-L4 and L4-L2/3 synaptic connections (Eggermann and Feldmeyer, 2009) probably by decreasing the open probability of presynaptic Ca^2+^ channels (Brown, 2010). The exclusive presence of M_4_Rs in layer 4 may serve to functionally define this layer in sensory cortices. This finding is, however, in marked contrast to immunohistochemical studies that show only weak M_4_R expression in layer 4 (see above).

A large fraction of slender-tufted L5A and thick-tufted L5B pyramidal cells respond to ACh with a rapid transient hyperpolarisation that is followed by a large and tonic depolarisation, as found for L2/3 pyramidal cells (Gulledge and Stuart, 2005; Gulledge et al., 2007; Eggermann and Feldmeyer, 2009; Nuñez et al., 2012; Dasari et al., 2017; see also **Figure 5C2**). This transient ACh-induced hyperpolarisations can be observed more frequently in L5 than in L2/3 pyramidal cells and are mediated by small-conductance, Ca^2+^-activated K^+^ channels (sK_Ca_ channels). The subsequent persistent depolarisation is due to an ACh-induced closure of voltage-gated K^+^ channels, K_ir_ channels and other K^+^ conductances; all these effects are the result of M_1_R activation (Gulledge and Stuart, 2005; Brown, 2010; Thiele, 2013; Dasari et al., 2017). L5B pyramidal cells with either corticocortical or subcortical projection targets (commisural, and corticopontine L5B pyramidal cells, that project to the contralateral cortex and the pons, respectively) have been shown to differ in their response to mAChR activation (Dembrow et al., 2010; see also Dembrow and Johnston, 2014 for a review). Following mAChR activation corticopontine but not commissural pyramidal cells showed a reduced current through hyperpolarization-activated, cyclic nucleotide-gated (HCN) channels and a high probability of shifting into a persistent AP firing mode. Almost all L6 pyramidal cells showed a strong, slowly depolarising M_1_R response (McCormick and Prince, 1986; Hedrick and Waters, 2015). In addition, in corticothalamic (CT) L6B pyramidal cells of the visual cortex a depolarising ACh response has been demonstrated that had a slow maintained mAChR- and a faster desensitizing nAChR-component (Sundberg et al., 2017; see also below).

Thus, the muscarinic ACh response shows a layer-specificity in two respects. First, the transient hyperpolarisation is found in L2/3 as well as L5A and L5B pyramidal cells albeit with different strength and frequency of occurrence between layers and cortical areas (Gulledge et al., 2007). Second, the persistent, tonic ACh response is depolarising in layers 2/3, 5 and 6 although the response amplitude and the response probability increases with cortical depth. Layer 4 in sensory cortices stands out in that ACh causes a persistent hyperpolarisation of L4 excitatory neurons, a result of the differential, layer-specific expression of mAChR subtypes. It should also be noted that despite this layer specificity, the ACh response is rather similar between different neocortical areas.

## Nicotinic Receptors

Nicotinic AChRs (nAChRs) are different from all other neuromodulator receptors because they are not coupled to G-proteins but form ligand-gated cation channels permeable to K^+^, Na^+^ and partially also Ca^2+^. There are 17 distinct subunits of ionotropic nAChRs, namely the α_1-10_, β_1-4_, γ, δ, and ε subunits. Nicotinic AChR channels contain five subunits and may be either homomeric or heteromeric [as pentameric combinations of α and β subunits mainly in the ratio (α)_2_:(β)_3_ although (α)_3_:(β)_2_ subunit combinations exist also]. The most abundant nAChR channel subtypes in the neocortex are the homomeric α_7_ and the heteromeric α_4_β_2_^∗^ channels, the latter of which is sometimes associated with an accessory, modulatory subunit (as indicated by the asterisk) such as the α_5_ subunit. The α_7_ nAChR channels show fast activation and a fast desensitization kinetics, are Ca^2+^-permeable and have only a low nicotine affinity; α_4_β_2_^∗^ nAChR currents have a slower onset, are more slowly desensitizing, less permeable to Ca^2+^ and show a high nicotine affinity. If α_4_β_2_^∗^ nAChRs contain also the accessory α_5_-subunit, the desensitization becomes even slower. ACh activates nAChRs either through volume transmission or via cholinergic synapses (Séguéla et al., 1993; Fucile, 2004; Xiao and Kellar, 2004; Dani and Bertrand, 2007; Gotti et al., 2007; see also Hedrick and Waters, 2015; Hay et al., 2016).

In the neocortex, six different nAChR subunits are expressed, namely the α_3_, α_4_, α_5_, α_7_, β_2_ and β_4_ subunits. The α_3_ mRNA is strongly and almost exclusively expressed in layer 4 while α_4_ mRNA is moderately and β_2_-subunit mRNA only weakly expressed in almost all layers. The α_5_ subunit is expressed at moderate levels in layer 6B but not at all or only weakly so in other neocortical layers. The α_7_ subunit shows a moderate to high expression in layers 1–3, 5, and 6 and no expression in layer 4. The β_4_ subunit mRNA shows a strong expression n layer 4 and moderate expression in all other cortical layers (Wada et al., 1989, 1990; Dineley-Miller and Patrick, 1992; Séguéla et al., 1993). It should be noted, however, that in none of these studies the cellular expression of the nAChR subunits was determined so that it is unclear whether the nAChRs are present in either presynaptic terminals of longe-range axons, interneurons or principal excitatory cells.

As found for mAChRs, the distribution of nAChRs is layer- and pyramidal cell type-specific. In both PFC and S1 barrel cortex, almost all L2/3 pyramidal cells show no nicotinic ACh response and therefore do not express nAChRs (Gil et al., 1997; Poorthuis et al., 2013a; Koukouli et al., 2017). In frontal cortex, however, Chu and coworkers recorded cholinergic EPSPs in L2/3 pyramidal cells. This may suggest that at least in some neocortical areas supragranular pyramidal cells are modulated by nAChRs (Chu et al., 2000). In marked contrast, all infragranular pyramidal cells express nAChRs.

**FIGURE 6:**
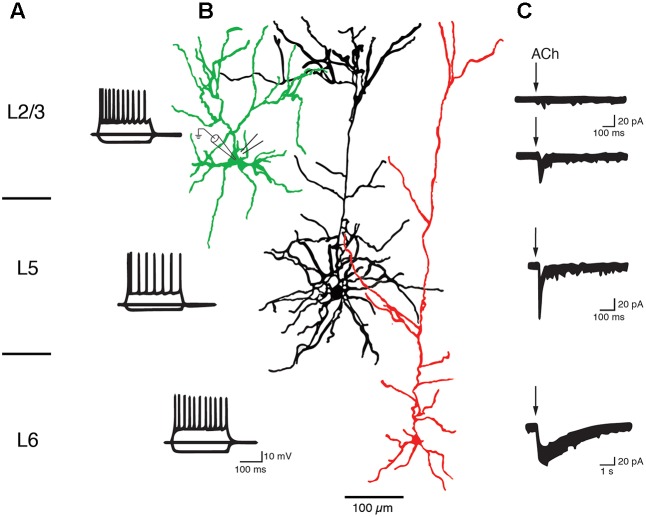
Layer-specific nAChR responses in pyramidal cells of the PFC. Current responses of L2/3, L5, and L6 PFC pyramidal cells to rapid ACh application. **(A)** AP firing pattern elicited by 300 ms current steps in PFC pyramidal cells of cortical layers 2/3, 5, and 6. **(B)** Morphological reconstructions of example L2/3, L5, and L6 pyramidal cells, in green, black and red, respectively. The recording (left) and puff pipette for rapid application) are shown at the soma of the L2/3 pyramidal cell **(C)** Response of pyramidal cells to brief applications of ACh. About 90% of L2/3 pyramidal cells did not display a nicotinergic ACh response (top trace). A small fraction (∼10%) of L2/3 and all L5 pyramidal cells showed rapid inward currents following ACh application, a hallmark of α_7_ nAChR-mediated currents. L6 pyramidal cells showed very slowly desensitizing ACh-induced currents that are mediated by α_4_β_2_α_5_ nAChRs (see text for details). After Poorthuis et al. (2013a) with permission from Oxford University Press.

Slender-tufted L5A pyramidal cells in S1 cortex respond to ACh application with a rapidly sensitizing inward current and are thus likely to express α_7_ nAChRs (Nuñez et al., 2012). Similarly, thick-tufted L5B pyramidal cells in the PFC express α_7_ nAChR as indicated by their low sensitivity to nicotine (Couey et al., 2007), fast nAChR response and block by a specific α_7_ nAChR antagonist (Poorthuis et al., 2013a; see also **Figure 6**). On the other hand, Hedrick and Waters recorded cholinergic EPSPs in L5 pyramidal cells that were elicited by optical stimulation of the basal forebrain and mediated by non-α_7_ (probably α_4_β_2_) nAChRs because they were blocked by a specific α_4_β_2_ nAChR antagonist. The nAChR-mediated EPSPs were prominent in primary motor (M1) and V1 cortex but rare in PFC (Hedrick and Waters, 2015). Slow ACh EPSPs in M_1_ L5 pyramidal cells could only be recorded in the soma and basal dendritic compartments; the apical dendrite and tuft were unresponsive to ACh. In another study a dual component nAChR response was recorded in L5 pyramidal cells of both frontal and somatosensory cortex that was mediated by both α_7_ and α_4_β_2_ receptors, with the latter becoming more prominent during prolonged ACh application (Zolles et al., 2009). These conflicting results may result from the fact that cholinergic EPSPs and whole cell responses are mediated by different nAChR subtypes as well as neocortical region-specific differences in the expression of nAChR subtypes.

In both L6A and L6B pyramidal neurons, ACh application induces a very slowly desensitizing inward current indicating the presence of α_4_β_2_^∗^ nAChR combined with the accessory α_5_ subunit that further slows down receptor desensitization (Kassam et al., 2008; Alves et al., 2010; Bailey et al., 2012; Poorthuis et al., 2013a,b; Hay et al., 2015; see also Sundberg et al., 2017). In addition, cholinergic EPSPs that were exclusively mediated by α_4_β_2_β_5_ nAChRs and devoid of a α_7_-component were also recorded in L6 pyramidal cells (Hay et al., 2016).

**FIGURE 7:**
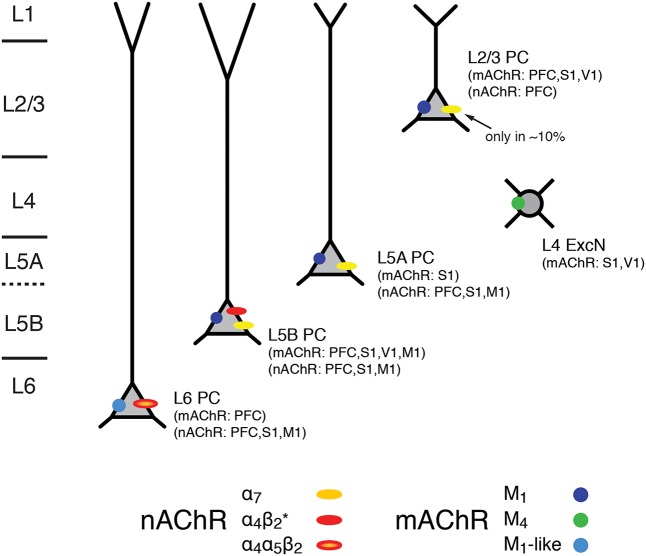
Expression of mAChRs and nAChRs in the neocortex. Schematic diagram of the layer- and cell-type specific distribution of nAChRs and mAChRs in the neocortex. Cortical layering is indicated on the left. Pyramidal cells (PC) in layers 2/3, 5A, 5B and 6 are shown; L5A are generally slender-tufted and L5B thick-tufted pyramidal cells. L4 excitatory neurons (L4 ExcN) include L4 spiny stellate, star pyramids and pyramidal cells. The different brain regions from which the mAChR and nAChR distribution were obtained are given in brackets.

Hence, the excitability of L5A, L5B, and L6 pyramidal cells is not only modulated by mAChRs alone but also via nAChRs that preferentially increase the activity of these deep-layer neocortical pyramidal neurons; only a small subset of L2/3 and no L4 excitatory neurons appear to express nAChRs. L6 pyramidal cells show a predominant expression of the slowly desensitizing α_4_β_2_α_5_ nAChRs which sets them apart from those in other cortical layers. The laminar and cell-specific distribution of these AChR classes is shown in a simplified schematic diagram in **Figure 7** (see also **Table 1**). The fact that both receptor classes act on very different time scales and at different agonist concentrations adds another level of complexity to the ACh modulation of neocortical signaling.

## Dopamine Receptors

Dopamine is involved in motor control and many higher cognitive functions such as attention, working memory, decision making, and reward. Receptors for dopamine fall into to groups, the D1-class receptors (D1 and D5) of which are mainly coupled to G_s_-proteins. D2-class receptors (D2, D3, and D4) on the other hand are coupled to G_i/o_ proteins. Via G_s_ proteins, D1Rs activate AC, increase intracellular cAMP levels which then results in the stimulation of PKA. PKA suppresses the activity of K_Ca_ channels that mediate the slow afterhyperpolarization (AHP) following an AP (Pedarzani and Storm, 1993; Satake et al., 2008; Yi et al., 2013). In addition, PKA reduces also the open probability of voltage-gated, slowly inactivating K^+^ currents (Dong and White, 2003) and K_ir_ channels (Dong et al., 2004). It has also been suggested that PKA enhances a persistent Na^+^ current (Yang and Seamans, 1996) or the rapidly inactivating Na^+^ current (Maurice et al., 2001). Furthermore, cAMP directly, i.e., independent of PKA, upregulates HCN channels (Pedarzani and Storm, 1995).

There is also evidence that particularly D5Rs but also D1Rs couple to G_q_ proteins. Their activation will result in an augmented PLC activity which will trigger intracellular IP_3_ production and intracellular Ca^2+^ release. This will potentiate Ca^2+^-dependent ion conductances such as K_Ca_ channels (for reviews see Beaulieu and Gainetdinov, 2011; Tritsch and Sabatini, 2012).

D2-class receptors on the other hand will decrease the AC activity and cause a reduction in intracellular cAMP levels resulting in a down-regulation of all cAMP-dependent enzymes and ligand- and voltage-gated ion channels. In addition, D2 receptors (D2R) activate K^+^ conductances and deactivate N- P/Q- and R-type Ca^2+^ channels via direct interaction with β/γ G-protein subunit complex (see **Figure 2**; Beaulieu and Gainetdinov, 2011; Tritsch and Sabatini, 2012).

In the neocortex, dopamine is released from dopaminergic afferents mostly from the ventral tegmental area (VTA). These afferents project throughout all layers of the frontal, cingulate and rhinal cortices but almost exclusively in deep cortical layers 5 and 6 of most other cortical areas including the M_1_, S1 and V1 cortex (Berger et al., 1991; Nomura et al., 2014). In primate neocortex the dopaminergic innervation is much more dense than in rodents and targets all layers in all cortical areas (Berger et al., 1991). Dopaminergic afferents have been shown to establish close appositions with the dendrites of callosally and nucleus accumbens projecting L5 pyramidal cells (i.e., both intracortical and pyramidal tract projecting neurons) and L2, L3, L5, and L6 pyramidal cells in both rat and primate prefrontal cortex (Krimer et al., 1997; Carr et al., 1999; Carr and Sesack, 2000) suggesting a spatially restricted dopamine release. However, the number of dopaminergic appositions is relatively low and the exact signaling mechanisms at these contacts are not known.

**FIGURE 8:**
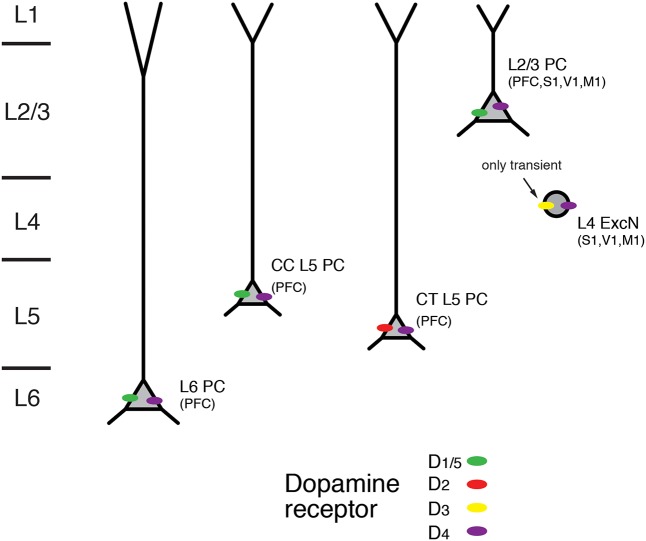
Expression of dopamine receptors in the neocortex. Schematic diagram of the layer- and cell-type specific distribution of dopamine receptor types in different pyramidal cell types in the neocortex. Data were obtained for pyramidal cells (PC) in layers 2/3, 5, and 6; CC and CT denote L5 PC with corticocortical and corticothalamic projection targets. L4 excitatory neurons (L4 ExcN) include L4 spiny stellate, star pyramids and pyramidal cells. Brain regions for which the receptor distribution were obtained are given in brackets. Apart from L4 ExcN all data are from functional, mainly electrophysiological studies (see text for details).

Studies of dopaminergic modulation have focussed mostly on pyramidal cells in layers 5 and 6 of the PFC because of the high density of dopaminergic afferents in this brain region and layers. Nevertheless, dopamine receptors have been found in all cortical layers and in many different cortical areas including sensory cortices (see **Figure 8**).

In accordance with the dense dopaminergic innervation of deep cortical layers, both D1R and D2R mRNA expression and immunoreactivity was stronger in layers 5 and 6 than in superficial or intermediate layers in the medial PFC (Weiner et al., 1991; Gaspar et al., 1995; Vincent et al., 1995; Santana et al., 2009; for a review see Santana and Artigas, 2017). D1R mRNA showed a particular abundance in deep layer 6 (i.e., layer 6B); on the other hand, expression of D2R was largely confined to layer 5 where it was higher than that of D1R (Santana et al., 2009). An analysis of the cellular distribution of D2R mRNA showed that it was present mostly in corticocortical (CC), CT and corticostriatal (CStr) projection neurons (Gaspar et al., 1995). In addition, using functional imaging of PKA activity Nomura and coworkers found wide-spread functional expression of D1/5Rs but also D2Rs throughout layers 2/3 and 5 of the frontal, parietal and occipital cortices (Nomura et al., 2014). In this study, only moderate regional and laminar-specific differences in the distribution of the different receptor subtypes were found.

D3R mRNA but no that of D1R or D2R has been detected in layer 4 of rodent S1 barrel cortex. Using receptor autoradiography and *in situ* hybridisation a transient but selective expression of this dopamine receptor type was found until the second postnatal week. D3R expression declined thereafter and was completely absent in the adult (Gurevich and Joyce, 2000; Gurevich et al., 2001). In addition, using immunocytochemistry D3R expression has been reported for pyramidal neurons in layers 3 and 5 of the somatosensory cortex and the PFC (Ariano and Sibley, 1994). Furthermore, D4R immunoreactivity has been shown in L2/3 and L5 pyramidal neurons of PFC, cingulate and parietal cortex as well as in L4 excitatory neurons in M1, S1 and V1 cortex (Mrzljak et al., 1996; Wedzony et al., 2000; Rivera et al., 2008; for a review see Tritsch and Sabatini, 2012).

In most *in vitro* studies in which presynaptic dopamine effects were blocked, dopamine increased the intrinsic excitability of deep layer PFC pyramidal neurons by depolarising the resting membrane potential and/or promoting a slow but long-lasting increase in the number of action potentials elicited by somatic depolarization (Yang and Seamans, 1996; Gulledge and Jaffe, 1998; Gulledge and Jaffe, 2001; Lavin and Grace, 2001; Seamans et al., 2001; Gao and Goldman-Rakic, 2003; Wang and Goldman-Rakic, 2004; Rotaru et al., 2007; Kroener et al., 2009; Moore et al., 2011; Seong and Carter, 2012; Happel et al., 2014; Gorelova and Seamans, 2015; for reviews see Tritsch and Sabatini, 2012; Xing et al., 2016). Generally, these effects are mediated by D1R activation and include an enhanced AP firing frequency, a block of K^+^ conductances and an increase in a persistent Na^+^ current; they are blocked by D1R antagonists and mimicked by D1R agonists. Furthermore, D1R activation has been reported to increase in the amplitude of glutamatergic EPSPs in PFC L2/3 pyramidal cells (Gonzalez-Islas and Hablitz, 2003). Here, the underlying mechanism is probably a G_s_-protein-induced phosphorylation of synaptic AMPA and NMDA glutamate receptors (via the AC-cAMP-PKA signaling pathway) that results in a potentiation of the activity both receptor types. Furthermore, a presynaptic D1R- and D2R inhibition of glutamatergic synaptic transmission in L3 pyramidal cells in primate PFC has been reported; this inhibition was found only for distal but not local synaptic inputs (Urban et al., 2002).

Recent studies have shown that dopaminergic modulation in layer 5 of the PFC may depend on the pyramidal cell type and its projection target (Gee et al., 2012; Seong and Carter, 2012; see also Dembrow and Johnston, 2014). CT pyramidal cells differed from CC PFC L5 pyramidal cells in that they had a larger HCN channel current and thick-tufted apical dendrites. While D1Rs were only expressed in thin-tufted putative CC pyramidal cells, D2Rs were present in thick-tufted CT pyramidal cells. An increase in excitability induced by D1R agonist application was found in thin-tufted pyramidal cells (Seong and Carter, 2012). Conversely, in thick-tufted pyramidal cells that projected to the thalamus *but not* to the contralateral cortex, D2R activation resulted in a L-type Ca^2+^ channel- and NMDAR-dependent afterdepolarisation and thus a higher excitability (Gee et al., 2012). This suggests that D2Rs are expressed only in CT L5 pyramidal cells. A D2R-mediated increase in the excitability of thick-tufted PFC L5 pyramidal cells was also observed in another study; here dopamine caused an increase in the AMPA receptor component of EPSPs elicited by layer 2/3 stimulation that led to burst-firing (Wang and Goldman-Rakic, 2004).

Thus, D1Rs are functionally expressed throughout cortical layers 2/3, 5, and 6, with a particularly high expression level in the latter. In contrast, D2Rs are almost exclusively confined to layer 5 and show a cell-specific expression in CT L5 pyramidal cells. It is not known whether the very heterogeneous population of L6 excitatory neurons (see **Figure 1**) shows a similar differential modulation by dopamine. Therefore, more studies on structurally identified neuron types in the different cortical layers are necessary to obtain a detailed picture of the cell-specific distribution of different dopamine receptor subtypes.

## Orexin/Hypocretin Receptors

Orexin/Hypocretin is a peptide that is synthesized in neurons of the lateral hypothalamic area. It plays a pivotal role in the regulation of wakefulness and arousal (for reviews see Sakurai, 2007, 2013; Alexandre et al., 2013; Richter et al., 2014; Kukkonen, 2017). Orexin-releasing neurons synthesize two peptides, orexin A and orexin B (also hypocretin 1 and 2). These peptides act on two G-Protein coupled receptors, the orexin 1 (OX1R; also HCRTR1) and orexin 2 (OX2R; also HCRTR2) receptor. While OX1R has a ∼100-fold higher binding affinity for orexin A than B, OX2R has a similar affinity for both orexins. The OX1R is mainly coupled to a G_q_ G-protein and causes an increase in intracellular Ca^2+^ (via PLC and IP_3_ activation; see above and **Figure 2**). OX2Rs are also coupled to G_i/o_-proteins and thus act by inhibiting K^+^ and Ca^2+^ currents. The distribution of mRNA for the OX1R and OX2R is markedly different and often complementary, suggesting that these receptors have distinct functional roles. While OX1R was only weakly expressed in the neocortex, a strong expression of OX2Rs has been found in neocortical layer 6. In addition, weak expression of OX2R has been reported to be present in layers 2/3 and in a few L5 pyramidal cells (Trivedi et al., 1998; Lu et al., 2000; Marcus et al., 2001; Cluderay et al., 2002).

Electrophysiological studies in the S1, V1, M1 and cingulate cortex have shown that in superficial layers of the neocortex orexin did not elicit a response at all and only a minute one in ∼10% of L5 pyramidal cells (Bayer et al., 2004), in line with the immunohistochemical and mRNA expression data. A substantial orexin-response was exclusively observed in L6B neurons where orexin B binds to the OX2R and causes a depolarisation by blocking K^+^ currents, a response that is potentiated by activation of α_4_β_2_α_5_ nAChRs (Bayer et al., 2004; Hay et al., 2015; Wenger Combremont et al., 2016a,b). No orexin-induced response was recorded in L6A neurons (Hay et al., 2015). It has been suggested that the main target neurons of orexin modulation in layer 6B are multipolar spiny neurons, indicating a cell-specific action of orexin (Wenger Combremont et al., 2016b). Excitatory L6B neurons innervate predominantly neurons in infragranular layers 5 and 6 (Clancy and Cauller, 1999; Marx and Feldmeyer, 2013). It has been proposed that one function of the orexin-sensitive L6B neurons is to recruit pyramidal neurons in the thalamorecipient layer 6A. Thus, thalamocortical signaling in layer 6A will be potentiated in an orexin-gated feedforward loop, and become more reliable (Hay et al., 2015). Remarkably, while almost all other neuromodulator systems show functional receptor distributions that extend through almost all layers of the neocortex, the OX2R stands out because it is found almost exclusively in layer 6B excitatory neurons. Therefore, OX2R can be considered as a specific marker for this layer.

While OX2R-mediated depolarisations have only been recorded in L6B neurons of S1, V1, M1 and cingulate cortex, the OX1R receptor appears to be more distributed throughout the cortical layers. It has been shown that in the PFC, orexin acting via OX1R and PKC can increase the excitability of PFC L2/3 and L5 pyramidal cells by inhibiting HCN channels and K^+^ conductances (Li et al., 2010; Yan et al., 2012). Thus in contrast to OX2R, OX1R is a less specific marker for cortical lamination.

## Conclusion

On the basis of the available data the expression pattern of neuromodulator receptors in the neocortex shows a high degree of layer- and cell-specificity (see **Figures 4**, **7**, **8** and **Table 1**). This is probably the result not of a layer-specificity *per se* but due to the fact that neurons with very distinct morphological properties (such as thick-tufted L5 pyramidal cells or L4 spiny stellate cells) are largely or even exclusively confined to a distinct layer.

Differences in the neuromodulator response could be the result of a virtual absence of a neuromodulatory receptor, its exclusive presence or changes in a receptor subtype in a layer and/or cell-type specific fashion. All neuromodulator systems described in this review fulfill at least one if not more of these criteria and may therefore serve to define cortical layers to some extent: An exclusive absence of a response was found for the adenosinergic system for which all superficial L2/3 pyramidal cells were shown to be unresponsive to adenosine while excitatory neurons in all other layers respond to adenosine with a hyperpolarisation. The only layer showing an orexin/hypocretin response is layer 6B. L4 excitatory neurons express the M_4_ mAChR while supra- and infra granular pyramidal cells show M_1_ mAChR responses. A similar situation was found for ACh acting on nicotinergic receptors where only L6 pyramidal cells showed an α_4_β_2_α_5_ nAChR response. Furthermore, several studies have demonstrated that the response to a neuromodulator is similar or even identical in different cortical areas, e.g., the tonic ACh-induced hyperpolarisation in L4 excitatory neurons found in the S1, A1 and V1 sensory cortices.

However, it has gradually become apparent, that the expression of neuromodulator receptors can vary between excitatory neurons in a defined layer. Excitatory neurons differ in their intra- and/or subcortical axonal targets, their dendritic morphology, electrophysiological properties and molecular make-up and thus may be subdivided in as many different cell types as GABAergic interneurons (Morishima and Kawaguchi, 2006; Morishima et al., 2011; Oberlaender et al., 2012; Narayanan et al., 2015; Zeisel et al., 2015; Tasic et al., 2016; Luo et al., 2017). Recent studies have demonstrated that this heterogeneity is often reflected in the neuromodulator receptor distribution and their effects (Dembrow et al., 2010; Gee et al., 2012; Seong and Carter, 2012; van Aerde et al., 2015). For the direction of future research it is therefore important that neuromodulation is investigated in identified neuron types, ideally in those for which the axonal projection pattern and target structures have been determined.

## Author Contributions

Both authors developed the paper concept, wrote the paper and drafted the figures.

## Conflict of Interest Statement

The authors declare that the research was conducted in the absence of any commercial or financial relationships that could be construed as a potential conflict of interest.
